# Enhancement of docosahexaenoic acid production by overexpression of ATP-citrate lyase and acetyl-CoA carboxylase in *Schizochytrium* sp.

**DOI:** 10.1186/s13068-020-01767-z

**Published:** 2020-07-21

**Authors:** Xiao Han, Zhunan Zhao, Ying Wen, Zhi Chen

**Affiliations:** State Key Laboratory of Agrobiotechnology and Key Laboratory of Soil Microbiology, Ministry of Agriculture, College of Biological Sciences, China Agricultural University, Beijing, 100193 China

**Keywords:** *Schizochytrium* sp., Docosahexaenoic acid, ATP-citrate lyase, Acetyl-CoA carboxylase, β-Galactosidase reporter system, Constitutive promoter

## Abstract

**Background:**

Docosahexaenoic acid (DHA) is an important omega-3 long-chain polyunsaturated fatty acid that has a variety of physiological functions for infant development and human health. Although metabolic engineering was previously demonstrated to be a highly efficient way to rapidly increase lipid production, metabolic engineering has seldom been previously used to increase DHA accumulation in *Schizochytrium* spp.

**Results:**

Here, a sensitive β-galactosidase reporter system was established to screen for strong promoters in *Schizochytrium* sp. Four constitutive promoters (*EF*-*1α*_*p*_, *TEF*-*1*_*p*_, *ccg1*_*p*_, and *ubiquitin*_*p*_) and one methanol-induced *AOX1* promoter were characterized by the reporter system with the promoter activity *ccg1*_*p*_> *TEF*-*1*_*p*_ > *AOX1*_*p*_ (induced) > *EF*-*1α*_*p*_ > *ubiquitin*_*p*_. With the strong constitutive promoter *ccg1*_*p*_, *Schizochytrium* ATP-citrate lyase (ACL) and acetyl-CoA carboxylase (ACC) were overexpressed in *Schizochytrium* sp. ATCC 20888. The cells were cultivated at 28 °C and 250 rpm for 120 h with glucose as the carbon source. Shake-flask fermentation results showed that the overexpression strains exhibited growth curves and biomass similar to those of the wild-type strain. The lipid contents of the wild-type strain and of the OACL, OACC, and OACL-ACC strains were 53.8, 68.8, 69.8, and 73.0%, respectively, and the lipid yields of the overexpression strains were increased by 21.9, 30.5, and 38.3%, respectively. DHA yields of the wild-type strain and of the corresponding overexpression strains were 4.3, 5.3, 6.1, and 6.4 g/L, i.e., DHA yields of the overexpression strains were increased by 23.3, 41.9, and 48.8%, respectively.

**Conclusions:**

Acetyl-CoA and malonyl-CoA are precursors for fatty acid synthesis. ACL catalyzes the conversion of citrate in the cytoplasm into acetyl-CoA, and ACC catalyzes the synthesis of malonyl-CoA from acetyl-CoA. The results demonstrate that overexpression of ACL and ACC enhances lipid accumulation and DHA production in *Schizochytrium* sp.

## Background

Docosahexaenoic acid (DHA, C22:6-∆4,7,10,13,16,19) is an omega-3 long-chain polyunsaturated fatty acid (LC-PUFA). As the principal omega-3 fatty acid in brain gray matter, DHA has neurotrophic and neuroprotective properties that are required for normal perinatal cortical maturation [1]. In addition, DHA supplementation improves human health by increasing cardioprotective, anti-inflammatory, and anticancer activities [2, 3]. DHA is therefore widely used as a nutritional supplement, often as a nutraceutical.

The conventional source of DHA is fish oil obtained from cold-water marine fish. Seasonal variation, overharvest, and population decline, however, prevent the steady supply of DHA that is required to meet the increasing market demands. Other commercial sources of DHA production are thraustochytrids, which are marine microorganisms [4]. *Schizochytrium* spp., as well as other thraustochytrids (such as species of *Thraustochytrium* and *Ulkenia*), are excellent DHA producers [5, 6]. *Schizochytrium* spp. can produce total fatty acids (TFAs) that represent up to 70% of the cell weight, with DHA representing 25–45% of TFAs [7, 8]. Owing to the increasing demand for DHA, many researchers have attempted to increase DHA production by *Schizochytrium* spp. [5, 6, 9]. To date, most studies of DHA production by *Schizochytrium* spp. have focused on the adaptive evolution of the strains [9]; on the optimization of medium composition including sources of carbon and nitrogen and the addition of inorganic salts and antioxidants [5, 6, 10, 11]; and on cultivation conditions and cultivation styles [12, 13]. Only a few studies have employed metabolic engineering to increase DHA accumulation in *Schizochytrium*. Yan et al. [14], for example, introduced the *Escherichia coli* acetyl-CoA synthase gene into *Schizochytrium* sp. TIO1101, which increased the biomass and TFA production of the resulting transformant by 29.9% and 11.3%, respectively. Introduction of an exogenous ω-3 desaturase gene into *Schizochytrium* sp. converted 3% docosapentaenoic acid (DPA) into DHA [15]. By increasing the number of active ACP domains of PUFA synthase, DHA productivity was increased by 1.8-fold in a recombinant *E. coli* expressing *Schizochytrium* PUFA biosynthetic genes [16]. These studies demonstrate that metabolic engineering can increase DHA production by *Schizochytrium* spp.

Metabolic engineering has also been used with the oleaginous yeast *Yarrowia lipolytica*, i.e., metabolic engineering efficiently increased the yeast’s production of total lipids and ω-3 PUFAs [17–20]. By rewiring the metabolic pathways of *Y. lipolytica*, researchers increased lipid accumulation > 60-fold, and caused lipid content to approach 90% of cell mass [18]. Compared to the 10–15% lipid content in wild-type (WT) *Y. lipolytica* [21], *Schizochytrium* spp. produces much higher lipid levels, and ω-3 PUFA DHA represents up to 45% of TFAs. Because the genome sequences of several strains of thraustochytrids (*Schizochytrium*, *Thraustochytrium*, and *Aurantiochytrium*) are now available [22–24], metabolic engineering should be an efficient way to rapidly increase their production of DHA and lipids.

Acetyl-CoA is precursor for fatty acid synthesis. ATP-citrate lyase (ACL) catalyzes the conversion of citrate and CoA into acetyl-CoA and oxaloacetate, along with the hydrolysis of ATP [25]. ACL is present in all eukaryotes except non-oleaginous yeasts. In animals and oleaginous basidiomycete yeasts, ACL is encoded by a single gene [26, 27]; in plants and some filamentous fungi, ACL usually consists of two subunits (ACL1 and ACL2) with homology to the N- and C-terminals of the animal ATP-citrate lyase polypeptide [28]. Fatty acid content was increased in *Y. lipolytica* by overexpression of ACL1 and ACL2 on a non-lipogenic medium in an obese strain [29] or overexpression of ACL from *Mus musculus* [30]. Acetyl-CoA carboxylase (ACC) catalyzes the synthesis of malonyl-CoA from acetyl-CoA, which is the rate-limiting step in fatty acid synthesis [21]. There are two types of ACCs in nature: in most bacteria and plant chloroplasts, ACC usually consists of multiple subunits, including the biotin carboxylase (BC), the biotin carboxyl carrier protein (BCCP), the α-carboxyltransferase (α-CT) and the β-carboxyltransferase (β-CT); but in mammals, fungi, and the cytoplasm of most plants, ACC is a single multifunctional polypeptide [31]. Overexpression of acetyl-CoA carboxylase in the presence of thioesterase in *E. coli* led to a sixfold increase in the rate of fatty acid synthesis [32]. ACC overexpression increased lipid content in *Y. lipolytica* and free fatty acid production in *S. cerevisiae* [21, 33].

To date, very few studies have attempted to enhance DHA production in *Schizochytrium* spp. through metabolic engineering. In this study, a sensitive β-galactosidase reporter system was established in *Schizochytrium* sp. to screen for strong promoters. Because a sufficient supply of acetyl-CoA and malonyl-CoA is a prerequisite for efficient lipid accumulation, *Schizochytrium* ACL and ACC were overexpressed under the strong constitutive promoter *ccg1*_*p*_ in *Schizochytrium* sp. ATCC 20888 to enhance lipid accumulation and DHA production.

## Results

### Developing a β-galactosidase reporter system in *Schizochytrium*

**Fig. 1:**
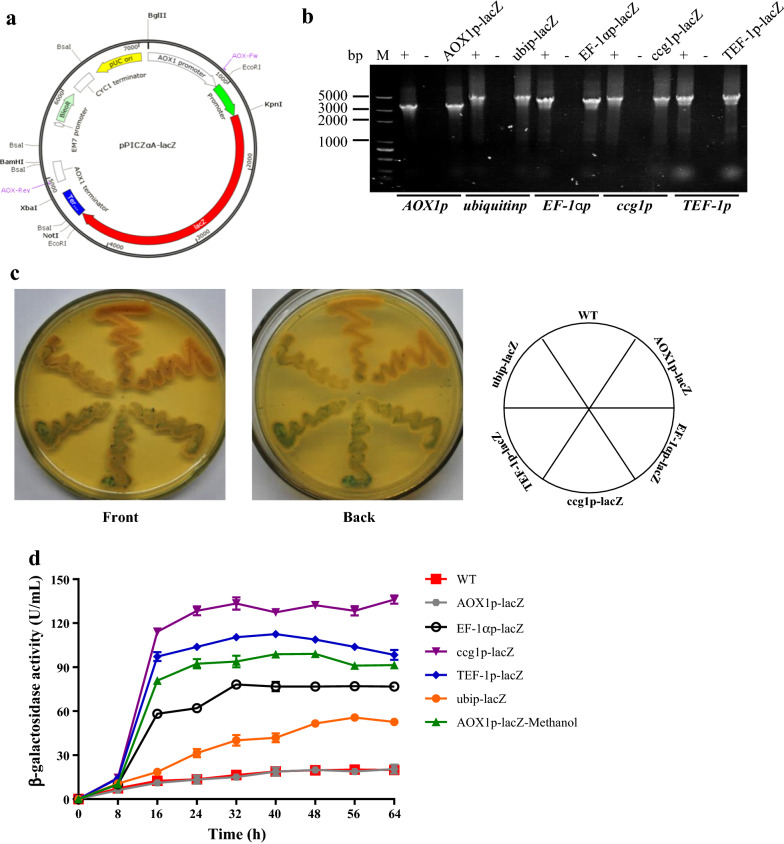
Development of a β-galactosidase reporter system and selection of strong promoters in *Schizochytrium* sp. **a** Construction of reporter plasmid pPICZαA-lacZ. **b** PCR verification of transformants. +: positive control, reporter plasmid was used as template; −: negative control, WT genomic DNA was used as template; AOX1p-lacZ, ubip-lacZ, EF-1αp-lacZ, ccg1p-lacZ, and TEF-1p-lacZ: the transformants of pPICZαA-AOX1p-lacZ, pPICZαA-ubiquitinp-lacZ, pPICZαA-EF-1αp-lacZ, pPICZαA-ccg1p-lacZ, and pPICZαA-TEF-1p-lacZ. **c** Phenotypes of the corresponding transformants. Cells were grown for 96 h on GPY plates with 40 μg/mL X-gal. **d** β-galactosidase enzymatic activities of the transformants. Cells were grown in seed medium for 64 h

Metabolic engineering involves the rewiring various metabolic pathways to redirect metabolic flux towards the synthesis of target compounds. As a consequence, the expression of relevant pathways must be strictly coordinated to achieve a balanced expression and to avoid metabolic bottlenecks [34]. It is therefore crucial that a reliable reporter system to monitor gene expression levels is established in *Schizochytrium*. The *E. coli* β-galactosidase structural gene *lacZ* has been widely used as a candidate reporter gene, providing convenient methods for qualitative colorimetric detection on agars with 5-bromo-4-chloro-3-indolyl β-d-galactopyranoside (X-gal) and quantitative β-galactosidase activity assays with *O*-nitrophenyl-β-d-galactopyranoside (ONPG) [35]. To determine whether the β-galactosidase reporter works in *Schizochytrium*, we constructed the reporter plasmid pPICZαA-ubiquitinp-lacZ, in which the *E. coli lacZ* gene was driven by a *ubiquitin* promoter–terminator system (Fig. 1a). pPICZαA containing *lacZ* without a *ubiquitin* promoter (termed pPICZαA-AOX1p-lacZ) was also constructed as a control plasmid. The corresponding transformants of *Schizochytrium* sp. ATCC 20888 were selected on glucose–peptone–yeast extract (GPY) plates with zeocin. DNA fragments of 5.0 and 3.3 kb were amplified from the genomic DNAs of the ubip-lacZ and AOX1p-lacZ transformants, respectively; the sizes of the fragments corresponded with the sizes of the *lacZ* expression cassettes (Fig. 1b), indicating that both plasmids were integrated into chromosomes.

The transformants of ubip-lacZ and AOX1p-lacZ grew normally on GPY agar. When the substrate X-gal was added to GPY plates, *Schizochytrium* sp. WT produced orange colonies with a very slight blue color, indicating that the WT possessed endogenous β-galactosidase with very low activity. AOX1p-lacZ produced colonies that were similar to those of the WT, while ubip-lacZ produced blue colonies (Fig. 1c). β-galactosidase enzymatic activity was much higher in the lysate of the ubip-lacZ transformant than in the AOX1p-lacZ transformant (Fig. 1d). These results show that, although *Schizochytrium* WT possesses endogenous β-galactosidase activity, β-galactosidase is able to serve as a sensitive reporter system in *Schizochytrium*.

### Selection of strong promoters in *Schizochytrium*

The use of four commonly used eukaryotic promoters (*EF*-*1α*_*p*_, *ccg1*_*p*_, *TEF*-*1*_*p*_, and *AOX1*_*p*_) in addition to *ubiquitin*_*p*_ was characterized in the β-galactosidase reporter system. In pPICZαA-AOX1p-lacZ, a methanol-induced *AOX1*_*p*_ [36] is present upstream of the *lacZ* gene. On X-gal plates, AOX1p-lacZ produced orange colonies like those of the WT, but the transformants with other promoters produced blue colonies, and the transformants with *ccg1*_*p*_ or *TEF*-*1*_*p*_ produced the bluest colonies. β-galactosidase activity in AOX1p-lacZ without methanol induction treatment was similar to that in the WT (Fig. 1c), indicating that *AOX1*_*p*_ is inactive without induction by methanol. The enzymatic activities driven by *ccg1*_*p*_, *TEF*-*1*_*p*_, *EF*-*1α*_*p*_, or *ubiquitin*_*p*_ were much higher than that of WT, with promoter activity of *ccg1*_*p*_ > *TEF*-*1*_*p*_ > *EF*-*1α*_*p*_ > *ubiquitin*_*p*_ (Fig. 1d). These findings indicate that the four promoters are strong, constitutively expressed promoters. When methanol was added to a 12-h culture of AOX1p-lacZ to a final concentration of 1% (vol/vol), the β-galactosidase activity increased substantially and remained at a high level, with the induction strength between *TEF*-*1*_*p*_ and *EF*-*1α*_*p*_, indicating that *AOX1*_*p*_ can be recognized by *Schizochytrium* RNA polymerase and induced by methanol. Therefore, *AOX1* promoter can serve as a methanol-induced promoter in *Schizochytrium*, although the induction time and strength require optimization.

### Construction of ACL and ACC-overexpression strains in *Schizochytrium* sp.

**Fig. 2:**
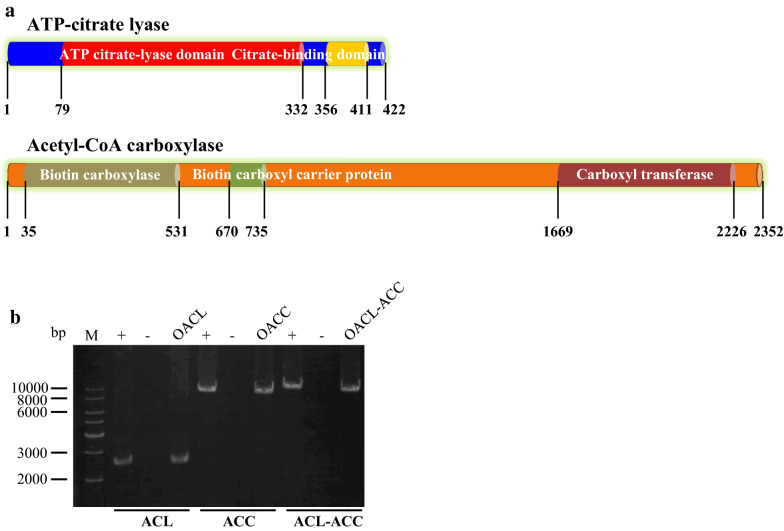
Overexpression of ATP-citrate lyase and acetyl-CoA carboxylase in *Schizochytrium* sp. **a** Schematic diagram of *Schizochytrium* sp. ACL and ACC. The proposed enzymatic domains are marked in different colors. **b** PCR verification of the transformants. +: positive control, the plasmid was used as template; −: negative control, WT genomic DNA was used as template; OACL, OACC, and OACL-ACC: genomic DNA from the transformants of pPICZαA-ACL, pPICZαA-ACC, and pPICZαA-ACL-ACC was used as template

In cytoplasm, ATP-citrate lyase converts intracellular citrate to acetyl-CoA in an ATP-dependent manner, and acetyl-CoA carboxylase catalyzes the synthesis of malonyl-CoA from acetyl-CoA [25]. Acetyl-CoA and malonyl-CoA are the precursors for fatty acids synthesis [21, 29]. A BLAST search of the *Schizochytrium* sp. CCTCC M209059 genome [22] revealed one putative ACL-encoding gene and one putative ACC-encoding gene (Additional file 2: Table S1). The putative ACL (422 aa) contains an ATP-citrate (pro-S)-lyase domain at the N-terminus, which is homologous to ATP-citrate lyase subunit 1, and a citrate-binding domain at the C-terminus, which is homologous to ATP-citrate lyase subunit 2 (Fig. 2a). Therefore, *Schizochytrium* ATP-citrate lyase functions as a single-subunit ACL. Like ACC in most fungi, ACC (2352-aa) in *Schizochytrium* is a single multifunctional polypeptide, containing a biotin carboxylase (BC) domain, a biotin carboxyl carrier protein (BCCP) domain, and a carboxyl transferase (CT) domain (Fig. 2a).

**Fig. 3:**
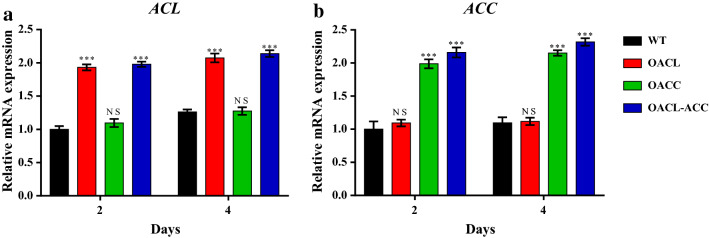
qRT-PCR analysis of the transcription levels of *ACL* and *ACC* in WT, OACL, OACC, and OACL-ACC. RNAs were isolated from WT, OACL, OACC, and OACL-ACC grown in fermentation media for 2 and 4 days. *P* values were determined by Student’s *t* test. ***P < 0.001; NS, not significant

To promote lipid biosynthesis and DHA accumulation in *Schizochytrium* sp. ATCC 20888, we developed transformants that overexpressed *Schizochytrium* ATP-citrate lyase and acetyl-CoA carboxylase. *ACL* and *ACC* genes were amplified from the cDNA of *Schizochytrium* sp. ATCC 20888 and were cloned separately or together into pPICZαA, in which the cloned gene was driven by the *ccg1* promoter and terminator (Additional file 1: Figure S1). After transformation, 2.8-, 8.7-, and 12.0-kb PCR fragments containing the corresponding expression cassettes (*ccg1*_*p*_-*ACL*-*ccg1*_*t*_, *ccg1*_*p*_-*ACC*-*ccg1*_*t*_, and *ccg1*_*p*_-*ACL*-*ccg1*_*t*_-*ccg1*_*p*_-*ACC*-*ccg1*_*t*_) were amplified from the genomic DNAs of pPICZαA-ACL, pPICZαA-ACC, and pPICZαA-ACL-ACC transformants (termed OACL, OACC, and OACL-ACC) (Fig. 2b), indicating that the plasmids were integrated into the chromosomes. The transcription levels of *ACL* and *ACC* were examined by qRT-PCR in WT, OACL, OACC, and OACL-ACC cultivated in fermentation broth for 2 and 4 d. Compared to WT, the expression of *ACL* were increased in OACL and OACL-ACC, and the expression of *ACC* were increased in OACC and OACL-ACC at both time points, indicating that transcription levels of *ACL* and *ACC* were increased in the corresponding overexpression strains (Fig. 3).

### ACL and ACC overexpression enhanced lipid accumulation

**Fig. 4:**
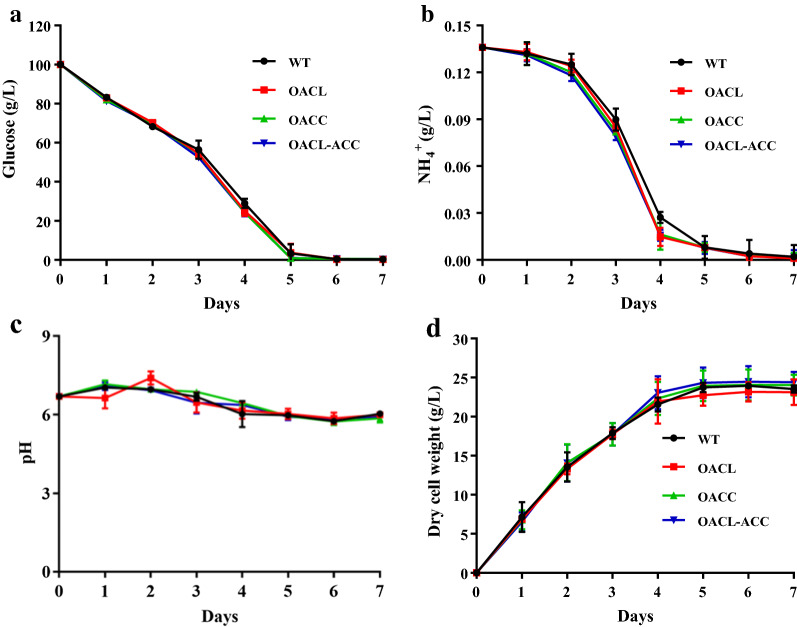
Time course of fermentation profiles of *Schizochytrium* sp. WT, OACL, OACC, and OACL-ACC. **a** Glucose (g/L); **b** NH_4_^+^ (g/L); **c** pH; **d** dry cell weight (DCW, g/L)

**Fig. 5:**
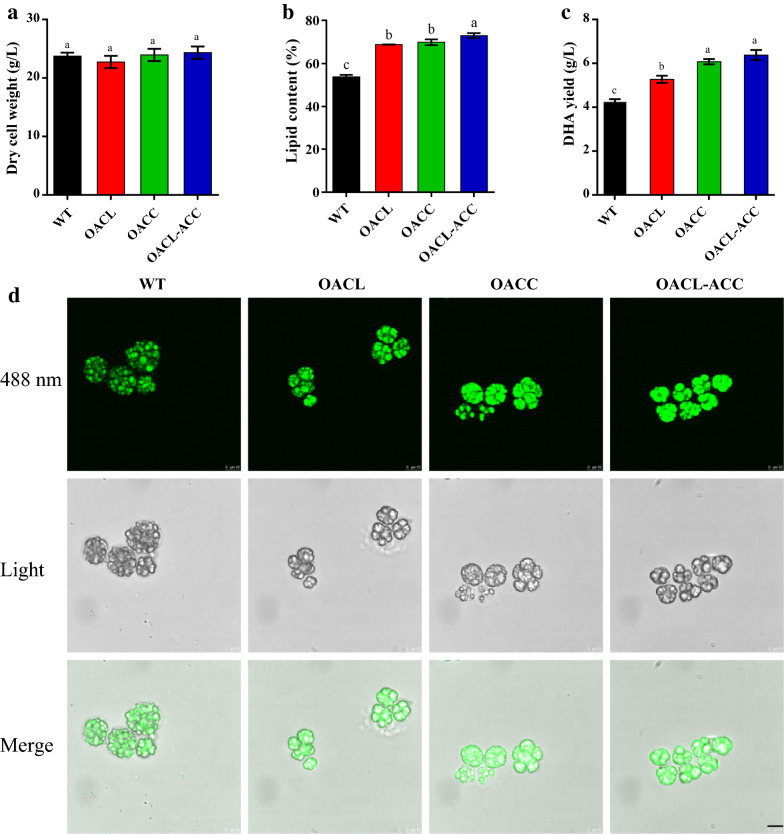
Effect of ACL and ACC overexpression on lipid accumulation and DHA production by *Schizochytrium* sp. **a** Dry cell weight (DCW, g/L). **b** Total lipids (% DCW). **c** DHA yield (g/L). Cells were cultured in fermentation medium for 120 h. **d** Imaging analyses of WT, OACL, OACC, and OACL-ACC. The 48-h cultured cells were stained with Nile red dye for neutral lipid staining. Scale bar, 10 µm. Error bars: SD from three independent experiments. Data were analyzed by one-way ANOVAs and Duncan’s multiple range tests in SPSS version 23.0. In **a**–**c**, columns with different lowercase letters are significantly different at *P* < 0.05

Shake-flask fermentation results showed that the WT strain and the overexpression transformants did not significantly differ in their consumption of carbon and nitrogen sources, pH values, or dry cell weights (DCW) (Fig. 4). DCW reached their maximum values on day 5, which were 23.7, 22.7, 23.9, and 24.3 g/L for WT, OACL, OACC, and OACL-ACC, and then decreased slightly with further cultivation (Figs. 4d, 5a). Thus, overexpression of ACL and/or ACC did not affect cell growth of *Schizochytrium*.

After 5 days of cultivation, lipid yields were lowest for the WT (12.8 g/L), highest for OACL-ACC (17.7 g/L), and intermediate for OACL (15.6 g/L), OACC (16.7 g/L) (Table 1). A similar pattern was evident for lipid content, i.e., lipid content was substantially higher in the overexpression strains than in the WT (Fig. 5b). Compared to the WT strain, the lipid yields were increased by 21.9% in OACL, by 30.5% in OACC, and by 38.3% in OACL-ACC. Microscopic observation revealed an increased intensity of lipid droplet staining in the overexpression strains (Fig. 5d). The findings indicated that overexpression of ACL and ACC increased lipid production in *Schizochytrium* sp., probably by increasing the supply of acetyl-CoA and malonyl-CoA.

**Table 1:** Fermentation characteristics of strains of *Schizochytrium* sp.

Strains	DCW (g/L)	Lipid yield (g/L)	Lipid content (%)	DHA yield (g/L)	DHA content (%)
WT	23.7 ± 0.6^a^	12.8 ± 0.5^d^	53.8^c^	4.3 ± 0.1^c^	36.4^b^
OACL	22.7 ± 1.0^a^	15.6 ± 0.4^c^	68.8^b^	5.3 ± 0.2^b^	36.2^b^
OACC	23.9 ± 1.1^a^	16.7 ± 0.7^b^	69.8^b^	6.1 ± 0.1^a^	37.6^a^
OACL-ACC	24.3 ± 1.1^a^	17.7 ± 0.3^a^	73.0^a^	6.4 ± 0.2^a^	37.9^a^

Cells were cultured in fermentation medium for 120 h. Data were analyzed by one-way ANOVAs and Duncan’s multiple range tests in SPSS version 23.0. Values with different lowercase letters are significantly different at *P* < 0.05

### ACL and ACC overexpression promoted DHA production

**Fig. 6:**
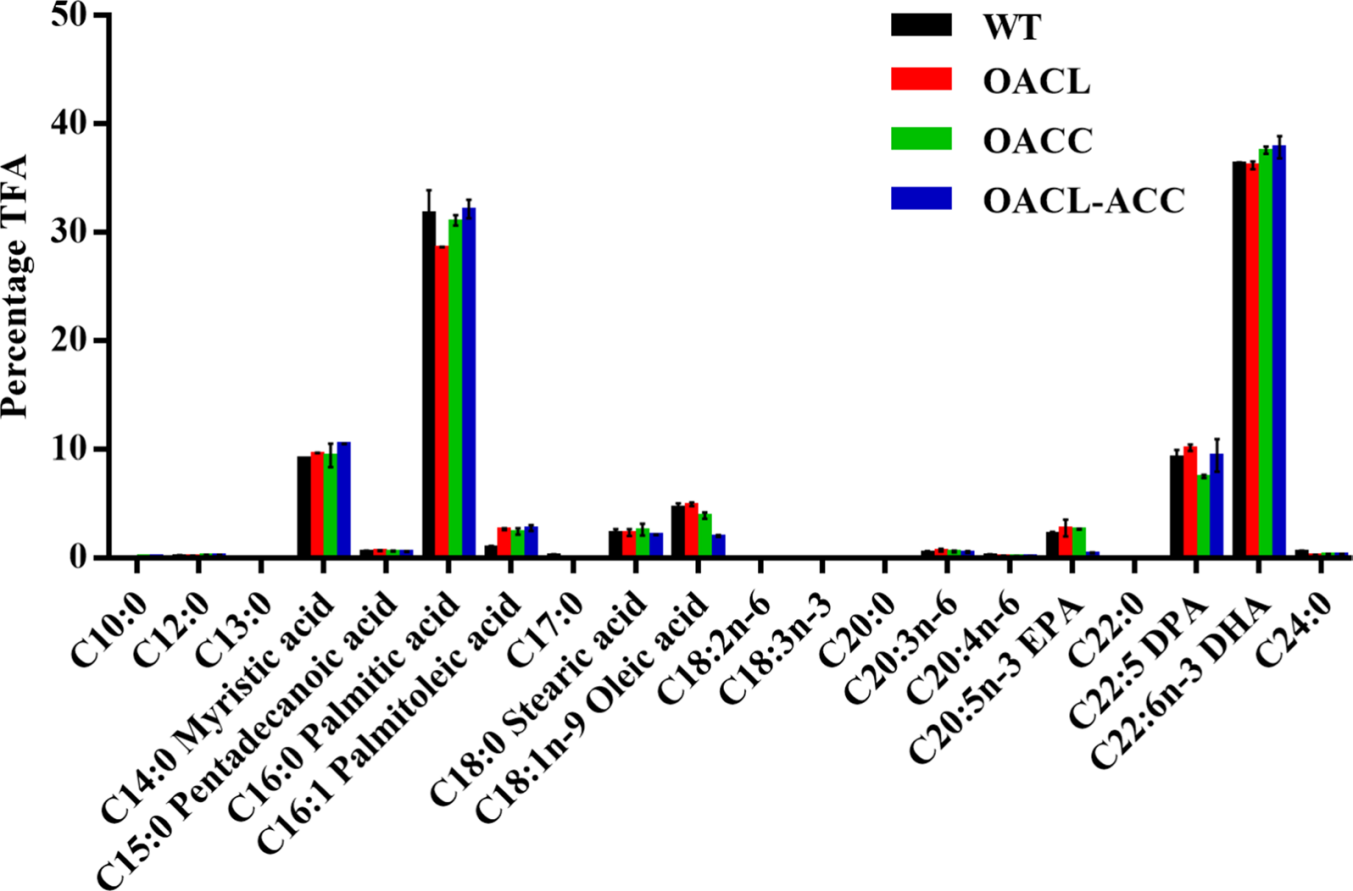
Effect of ACL and ACC overexpression on fatty acid composition (TFA, %) of *Schizochytrium* sp. Cells were cultured in fermentation medium for 120 h

Gas chromatography analysis showed that the main fatty acid components of *Schizochytrium* sp. ATCC 20888 were DHA, palmitic acid (C16:0), myristic acid (C14:0), and docosapentaenoic acid (DPA, C22:5) (Fig. 6). Overexpression of ACL in *Schizochytrium* sp. WT did not significantly affect the percentage of TFAs represented by DHAs (36.4% for the WT, and 36.2% for OACL) (Fig. 6), but slightly decreased the percentage represented by palmitic acid and slightly increased the percentage represented by C16:1. In ACC-overexpression strains, the percentage of TFAs represented by DHAs increased slightly for OACC (37.6%) and OACL-ACC (37.9%), and the percentage represented by oleic acid and DPA decreased slightly (Fig. 6). Compared to the DHA yield of the WT (4.3 g/L), the DHA yields of OACL, OACC, and OACL-ACC strains were 5.3, 6.1, and 6.4 g/L, increased by 23.3, 41.9, and 48.8%, respectively (Fig. 5c). These results indicated that overexpression of ACL and ACC greatly increased DHA production in *Schizochytrium* sp. ATCC 20888.

## Discussion

In this study, a sensitive β-galactosidase reporter system in *Schizochytrium* was developed and used to compare the strengths of some commonly used eukaryotic promoters. Although endogenous β-galactosidase is present in *Schizochytrium*, the reporter system was able to detect different levels of β-galactosidase activity with the LacZ-reporter driven by different promoters. *ccg1*_*p*_, *TEF*-*1*_*p*_, *EF*-*1α*_*p*_, and *ubiquitin*_*p*_ promoters are constitutive promoters and the *AOX1*_*p*_ promoter is inducible by methanol in *Schizochytrium*. More work can be carried out for subsequent research, such as screening for more endogenous constitutive promoters with different expression intensities and optimization of the induction conditions for the methanol-induced *AOX1*_*p*_ promoter. The β-galactosidase reporter system will facilitate characterization of novel genetic elements and will help identify promoters for fine-tuning gene expression in *Schizochytrium*.

Acetyl-CoA and malonyl-CoA are precursors for fatty acid synthesis [37]. Previous studies have shown that increasing the substrates supply significantly enhanced the synthesis of fatty acids and lipids in bacterium, yeast and fungi [30, 32, 38]. In the study, lipid and DHA production were greatly increased by overexpression of ACL and ACC. ACL converts intracellular citrate to acetyl-CoA [29], and ACC catalyzes the synthesis of malonyl-CoA from acetyl-CoA [21]. It follows that overexpression of ACL and ACC evidently promoted production of intracellular acetyl-CoA and malonyl-CoA, resulting in enhanced biosynthesis of fatty acids, which in turn promoted lipid accumulation. The percentage of TFAs represented by DHAs in ACL-overexpression strain was similar to that in the WT, indicating that overexpression of the *ACL* gene led to increased intracellular acetyl-CoA pools, which promoted the production of both saturated fatty acids and polyunsaturated fatty acids. In ACC-overexpression strains, the percentage of TFAs represented by DHAs improved slightly, while the percentage of TFAs represented by oleic acid and DPA decreased slightly. Thus, the increased malonyl-CoA pool in ACC-overexpression strains increased DHA production more than saturated fatty acid production. The findings suggested that the supply of malonyl-CoA is the limiting factor of DHA overproduction in *Schizochytrium*. In oleaginous microorganisms, efficient fatty acid synthesis requires not only an abundant supply of acetyl-CoA and malonyl-CoA, but also an ample NADPH supply [38, 39]. Therefore, combining this strategy with other strategies rewiring the metabolic flux of *Schizochytrium* towards precursors and NADPH accumulation might significantly improve its lipogenesis capability and DHA productivity.

*Schizochytrium* spp. are excellent producers of ω-3 PUFA: they can synthesize DHA de novo and DHA represents up to 45% of TFAs [7, 8]. To date, most studies of *Schizochytrium* spp. have focused on the optimization of fermentation media and cultivation conditions and the adaptive evolution of the strains [5, 6, 9–12]. Very few studies have employed metabolic engineering to improve DHA production of *Schizochytrium* spp. [14–16], mainly because the genetic background of *Schizochytrium* remains poorly understood. Metabolic engineering has been proven to be a highly efficient way to increase total lipids and ω-3 PUFA accumulation in the yeast *Y. lipolytica* [17–20]. In the current investigation, we demonstrated that metabolic engineering is an efficient way for increasing lipid accumulation and DHA production in *Schizochytrium*. The study provided a sensitive reporter system to monitor gene expression levels in *Schizochytrium* and a genetically engineered *Schizochytrium* sp. for industrial production of DHA.

## Conclusions

A strain of *Schizochytrium* sp. ATCC 20888 that overexpressed ACL and ACC under the strong constitutive promoter *ccg1*_*p*_ was constructed and thereby produced high quantities of DHA. Under shake-flask culture conditions, OACL, OACC, and OACL-ACC strains attained a dry cell weight of 22.7, 23.9, and 24.3 g/L, respectively. Compared to the WT, total lipid content of OACL, OACC, and OACL-ACC strains reached 68.8, 69.8, and 73.0%, respectively, and the lipid yields of the overexpression strains were increased by 21.9, 30.5, and 38.3%, respectively. A final DHA yield of 6.4 g/L in OACL-ACC was achieved, which was 48.8% higher than that of the WT. Next, fermentation control of OACL-ACC in fermentors will be optimized to make it more suitable for industrial application.

## Methods

### Microorganisms and culture conditions

Strains and plasmids used in the study are listed in Table 2. Media and growth conditions of *Schizochytrium* sp. were according to Ling et al. [5] with modifications. *Schizochytrium* sp. was cultured at 28 °C on solid GPY medium containing per liter 20 g of glucose, 10 g of peptone, 5 g of yeast extract, 20 g of sea crystal, and 20 g of agar. Transformants were selected and cultured on GPY supplemented with 40 μg/mL zeocin. For lipid and DHA production, 250-mL flasks containing 50 mL of seed medium (containing per liter 30 g of glucose, 10 g of peptone, 5 g of yeast extract, and 20 g of sea crystal) were inoculated with *Schizochytrium* sp. cells and incubated for 24 h at 28 °C on a rotary shaker (230 rpm). The seed culture was inoculated at 5% (vol/vol) into 50 mL of fermentation medium (containing per liter 100 g of glucose, 5 g of yeast extract, 3.94 g of NaCl, 0.264 g of KCl, 0.5 g of (NH_4_)_2_SO_4_, 1 g of KH_2_PO_4_, 1.43 g of MgSO_4_, 0.04 g of CaCl_2_, 10 g of sodium glutamate, 0.001 g of vitamin B_1_, and 0.001 g of vitamin B_12_) and was then incubated at 28 °C on a rotary shaker (250 rpm) for 168 h. For qualitative colorimetric and quantitative detection of β-galactosidase, cells were cultured on GPY plates with 40 μg/mL X-gal and in seed medium, respectively. *E. coli* was grown in LB medium at 37 °C [35].

**Table 2:** Strains and plasmids used in this study

Strain or plasmid	Description	Source or reference
*Schizochytrium* sp.		
ATCC 20888	Wild-type strain (WT)	American Type Culture Collection
AOX1p-lacZ	WT strain carrying pPICZαA-AOX1p-lacZ	This study
ubip-lacZ	WT strain carrying pPICZαA-ubiquitinp-lacZ	This study
TEF-1p-lacZ	WT strain carrying pPICZαA-TEF-1p –lacZ	This study
EF-1αp-lacZ	WT strain carrying pPICZαA-EF-1αp-lacZ	This study
ccg1p-lacZ	WT strain carrying pPICZαA-ccg1p-lacZ	This study
OACL	ACL overexpression strain	This study
OACC	ACC overexpression strain	This study
OACL-ACC	ACL and ACC co-overexpression strain	This study
*E. coli*		
JM109	General cloning host for plasmid manipulation	Laboratory stock
Plasmids		
pPICZαA	Yeast expression vector	[42]
pPICZαA-AOX1p-lacZ	*lacZ* reporter vector using *AOX1* promoter and terminator	This study
pPICZαA-ubiquitinp-lacZ	*lacZ* reporter vector using *ubiquitin* promoter and terminator	This study
pPICZαA-TEF-1p-lacZ	*lacZ* reporter vector using *TEF*-*1* promoter and terminator	This study
pPICZαA-EF-1αp-lacZ	*lacZ* reporter vector using *EF*-*1α* promoter and terminator	This study
pPICZαA-ccg1p-lacZ	*lacZ* reporter vector using *ccg1* promoter and terminator	This study
pPICZαA-ACL	ACL overexpression vector based on pPICZαA	This study
pPICZαA-ACC	ACC overexpression vector based on pPICZαA	This study
pPICZαA-ACL-ACC	ACL and ACC co-overexpression vector based on pPICZαA	This study

### Construction of β-galactosidase reporter plasmids and of ACL-, and ACC-overexpression plasmids

To construct β-galactosidase reporter plasmids, a 3075-bp fragment containing the coding sequence of the *lacZ* gene was amplified from pMC1403 [35] by PCR using primer pair lac-Fw and lac-Rev (Additional file 2: Table S2). The promoters and terminators of *EF*-*1α* [40] and *ubiquitin* [15] were amplified from *Schizochytrium* sp. ATCC 20888; *ccg1* promoter and terminator were amplified from the *Neurospora* expression vector pCCG.N-3xMyc [41]; and the *TEF*-*1* promoter and *CYC*-*1* terminator were amplified from the yeast expression vector pPICZαA [42] using the primer pairs listed in Additional file 2: Table S2. After purification, the *lacZ* gene was digested with *Kpn*I/*Not*I, the promoters were digested with *Eco*RI/*Kpn*I, and the terminators were digested with *Not*I/*Xba*I; the digested gene, promoters, and terminators were then simultaneously ligated into *Eco*RI/*Xba*I-digested pPICZαA to generate reporter plasmids. Ligation reactions were performed overnight at 16 °C using T4 DNA Ligase (TaKaRa, Japan).

To construct the ACL overexpression plasmid, a 1269-bp fragment of the *ACL* gene was amplified from *Schizochytrium* sp. ATCC 20888 cDNA. After purification, the *Kpn*I/*Not*I-digested *ACL* gene, *Eco*RI/*Kpn*I-digested *ccg1*_*p*_, and *Not*I/*Xba*I-digested *ccg1*_*t*_ were simultaneously ligated into *Eco*RI/*Xba*I-digested pPICZαA to generate the overexpression plasmid pPICZαA-ACL. For construction of the ACC overexpression plasmid, a 7059-bp *ACC* fragment was amplified from the same cDNA and purified. The *ACC* gene and the *ccg1* promoter and terminator were inserted into *Eco*RI-digested pPICZαA to generate pPICZαA-ACC using the Seamless assembly cloning kit (Clone Smarter, USA) following the manufacture’s protocol. To construct the ACL and ACC co-overexpression plasmid, the 2611-bp *ccg1*_*p*_-*ACL*-*ccg1*_*t*_ expression cassette was amplified from plasmid pPICZαA-ACL and was inserted into *Eco*RI-digested pPICZαA-ACC to generate pPICZαA-ACL-ACC by Seamless assembly cloning.

### Transformation of *Schizochytrium* sp.

Transformation of *Schizochytrium* was performed as described previously with modification [15]. *Schizochytrium* sp. cells were cultured in seed medium for 24 h to the logarithmic growth phase, and were harvested by centrifugation (5900*g*, 4 °C, 10 min) (HITACHI CF16RXII, Japan), washed with ice-cold sterile water, washed with 1 M sorbitol, and then suspended in 1 M sorbitol. The plasmids were linearized with restriction enzyme *Bam*HI before transformation. The competent cells and 5 μg of linearized plasmid DNA were placed in a 0.1-cm-gap cuvette. The parameters of electroporation were 0.75 kV, 200 Ω, 50 μF, twice. After electroporation, 1 mL of seed medium was added to the mixture, which was incubated at 28 °C for 4 h. The transformants were spread on GPY plates with 40 μg/mL zeocin and grown at 28 °C.

### Genomic PCR analysis of transformants

Genomic DNAs of putative transformants were extracted according to Lippmeier et al. [43]. To confirm *Schizochytrium* sp. transformants, the incorporation of the expression cassette into the genome was verified by PCR using primers AOX-Fw and AOX-Rev (Additional file 2: Table S2). PCR reactions were set up using *Taq* DNA polymerase (TaKaRa, Japan) following the manufacture’s protocol. PCR amplification parameters were as follows: 5 min at 95 °C; followed by 30 cycles of 50 s at 95 °C, 50 s at 55 °C, and 5 min at 72 °C; and a final extension for 10 min at 72 °C.

### β-Galactosidase activity assay

*Schizochytrium* sp. cells cultured in seed medium were collected by centrifugation at the indicated time, washed with phosphate buffer saline solution (PBS, 38.7 mM Na_2_HPO_4_∙12H_2_O, 11.3 mM NaH_2_PO_4_∙2H_2_O, and 150 mM NaCl), resuspended in 1 mL PBS, and disrupted with Mini Bead Beater (Biospec Mini-Bead-Beater-16 Model 607EUR, USA) for 50 s each for 5 times. After centrifugation, 500 μL of supernatant was transferred to a new tube. A 500-μL volume of buffer Z (60.0 mM Na_2_HPO_4_∙12H_2_O, 39.7 mM NaH_2_PO_4_∙2H_2_O, 10.0 mM KCl, 1.0 mM MgSO_4_∙7H_2_O, and 2.7 mL/L β-mercaptoethanol) and 200 μL of 13.3 mM ONPG were added to the supernatant, and the mixture was incubated at 37 °C for 15 min; the reaction was stopped by adding 500 μL of 1 M Na_2_CO_3_. The OD_420_ was recorded with an ultraviolet spectrophotometer for the determination of β-galactosidase activity. The amount of enzyme that releases 1 µmol of ONP per minute is defined as one unit of enzyme activity.

### Determination of dry cell weight, pH and glucose and nitrogen concentrations

Determination of dry cell weight was performed as described previously with modification [10]. A 40-mL volume of fermentation broth was centrifuged at 5900*g* for 10 min, and dry cell weight was determined after freeze-drying for 24 to 48 h to a constant weight. For measurement of pH, glucose and nitrogen concentrations, 1 mL of broth was centrifuged (Heraeus BIOFUGE pico, Germany) at 13,523*g* for 10 min, and the supernatant was used for determination. The pH was measured by a laboratory pH meter (METTLER TOLEDO FiveEasy, Switzerland). The concentration of glucose was determined by the 3,5-dinitrosalicylic acid (DNS) method [44, 45]. NH_4_^+^ concentration was measured by the indophenol blue spectrophotometric method [46].

### Microscopic analysis

Nile red staining of cells was conducted as described previously with modification [18]. A 1-mL volume of a culture grown in fermentation medium for 48 h was collected by centrifugation, washed twice with PBS solution, and resuspended in 1 mL of PBS. Cells were stained with Nile red dye (0.5 mg/L) and were incubated for 5 min in the dark. Fluorescence images were captured with a LEICA TCS SP8 microscope equipped with an oil immersion objective (×1000 magnification).

### Lipid extraction and fatty acid composition analysis

Lipids were extracted as described previously with some modification [47–49]. About 0.3 g of a freeze-dried *Schizochytrium* sp. pellet was mixed with 6 mL of 4 M HCl for 30 min and then incubated in boiling water for 8 min before 16 mL of methanol/chloroform (1:1, vol/vol) was added. The preparation was mixed vigorously, and then centrifuged at 129*g* for 10 min. The lower phase was transferred to a pre-weighed glass tube and evaporated under a stream of nitrogen.

Fatty acid methyl esters (FAMEs) were prepared according to Ren et al. [50] with some modifications. About 30 mg of lipid sample was transferred to a glass tube before 1 mL of internal standard (methyl nonadecanoate, C19:0, 1 mg/mL) and 1 mL of 0.5 M KOH in methanol were added; the mixture was incubated at 65 °C in a water bath for 15 min. After the mixture had cooled to room temperature, 2.1 mL of methanol and 0.9 mL of 45% BF3–ether were added to the tube, which was incubated at 65 °C for 5 min. Then 1 mL of hexane and 2 mL of saturated sodium chloride solution were added; the preparation was mixed vigorously and allowed to stand for 10 min. The upper layer of the solution was transferred to a new tube and used for analysis of fatty acid composition. FAMEs were separated by gas chromatography (WUFENG GC522) with an Agilent J & W DB23 capillary column (30 m × 0.25 mm i.d.). Nitrogen was used as the carrier gas at a flow rate of 2 mL/min. The injector was at 250 °C. The column temperature was increased from 150 to 200 °C at the rate of 5 °C per min, was kept at 200 °C for 1 min, was then raised to 230 °C at the rate of 4 °C per min, and was maintained at 230 °C an additional 9 min.

### RNA preparation and quantitative real-time PCR analysis (qRT-PCR)

*Schizochytrium* sp. cells cultured in fermentation medium were collected at 2 and 4 days, frozen in liquid nitrogen, and ground to fine powder. Total RNA was extracted with TRIzol reagent (Tiangen, China) according to the manufacturer’s protocol. cDNA was synthesized by M-MLV (RNase H^−^; TaKaRa) with oligo-dT18 from 4 µg of total RNA. qRT-PCR analysis was performed using FastStart Universal SYBR Green Master (ROX) with primers listed in Additional file 2: Table S2. PCR included a 10-min preincubation at 95 °C, followed by 40 cycles of denaturation at 95 °C for 10 s, and annealing and extension at 60 °C for 30 s. The relative expression levels were determined according to the comparative Ct method, using actin as the internal control.

### Statistical analysis

All experiments were performed with three biological replicates. Statistical analyses were performed using one-way ANOVAs and Duncan’s multiple range tests or two-tailed Student’s *t*-tests. And it was considered indicative of statistical significance at *p* < 0.05.

## Supplementary information

**Additional file 1: Figure S1.** Physical map of overexpression plasmid pPICZαA-ACL.

**Additional file 2: Table S1.** The open reading frames of *ACL* and *ACC* genes in *Schizochytrium* sp. **Table S2.** Primers used in this study.

## Data Availability

All data supporting the conclusions of this article are included in the manuscript and in the additional information.

## Abbreviations

ACC: Acetyl-CoA carboxylase
ACL: ATP-citrate lyase
DCW: Dry cell weight
DHA: Docosahexaenoic acid
DNS: 3,5-Dinitrosalicylic acid
DPA: Docosapentaenoic acid
FAMEs: Fatty acid methyl esters
LC-PUFA: Long chain polyunsaturated fatty acid
ONPG: *O*-Nitrophenyl-β-d-galactopyranoside
PBS: Phosphate buffer saline
TFAs: Total fatty acids
WT: Wild type
X-gal: 5-Bromo-4-chloro-3-indolyl β-d-galactopyranoside
