# Supplementary figures and images for: Enhancement of docosahexaenoic acid production by overexpression of ATP-citrate lyase and acetyl-CoA carboxylase in Schizochytrium sp
Source: Biotechnol Biofuels. 2020 Jul 21;13:131. doi: 10.1186/s13068-020-01767-z (PMC7372839; doi:10.1186/s13068-020-01767-z)

## Slide 1
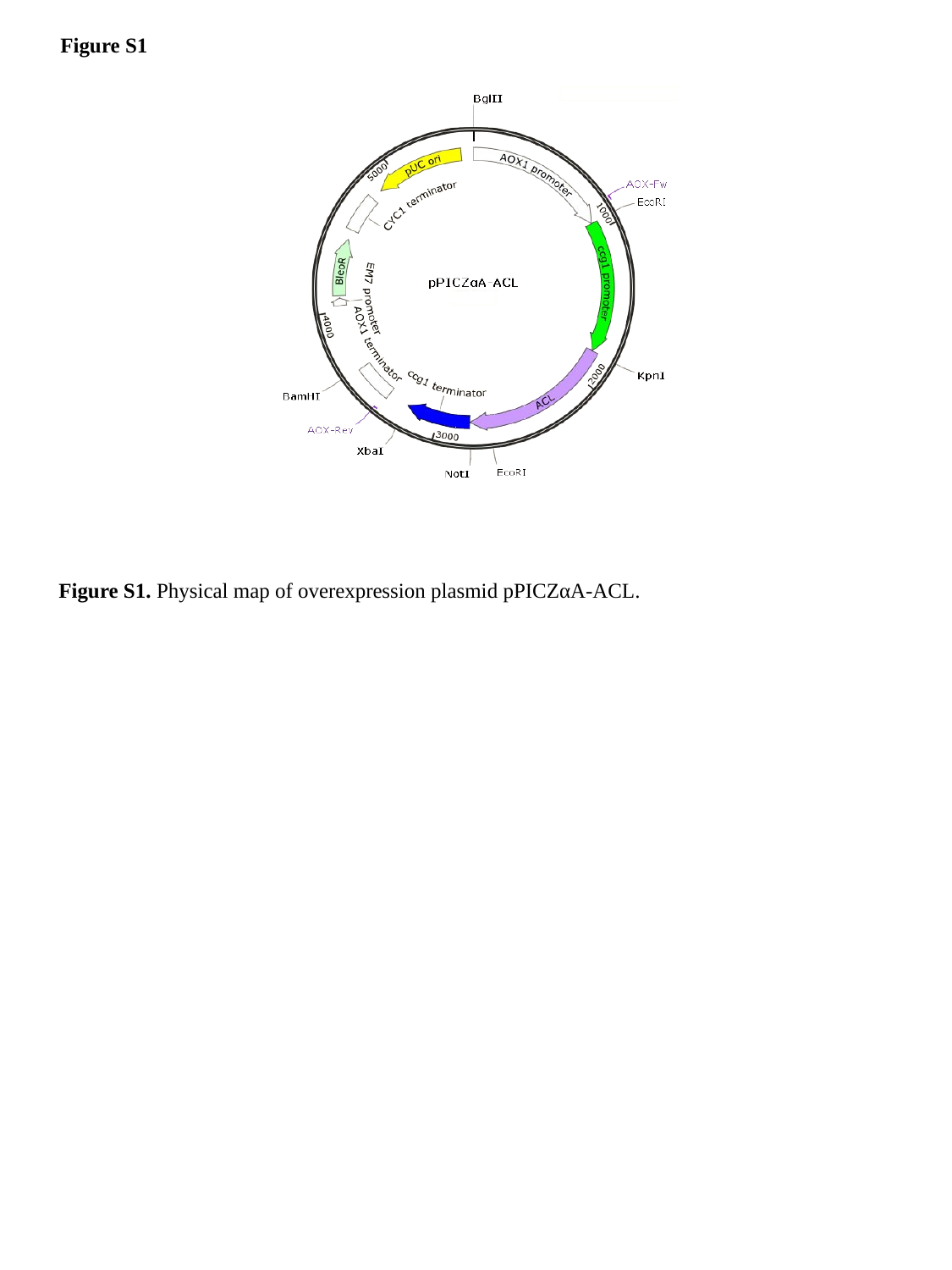

Figure S1
Figure S1. Physical map of overexpression plasmid pPICZαA-ACL.

Supplement: Supplementary file 1 — Additional file 1: Figure S1. Physical map of overexpression plasmid pPICZαA-ACL. [file 13068_2020_1767_MOESM1_ESM.pptx]
