# Supplementary material for: Enhancement of docosahexaenoic acid production by overexpression of ATP-citrate lyase and acetyl-CoA carboxylase in Schizochytrium sp
Source: Biotechnol Biofuels. 2020 Jul 21;13:131. doi: 10.1186/s13068-020-01767-z (PMC7372839; doi:10.1186/s13068-020-01767-z)
Supplement: Supplementary file 2 — Additional file 2: Table S1. The open reading frames of ACL and ACC genes in Schizochytrium sp. Table S2. Primers used in this study. [file 13068_2020_1767_MOESM2_ESM.docx]

**Table S1 The open reading frames of *ACL* and *ACC* genes in *Schizochytrium* sp.**

| **Name** | **Sequence (5’-3’)** |
| --- | --- |
| *ACL* | ATGGGGCCTAGGCCTACCGCAAACGAAACGTCATTTTCGCATCACTTGAC  CTGCTGTGTAGCTAAATTTGTAGATCTGCGCAACCCAACTTCTGACGTCG  GACGTCGTCTTTCGACCCTAGATCTCAAACGCGGAGCAATAAGAGTTAGT  AGAAGCGCAGCGCATTGCACCACGCAGCTTAGCATCGCATCACATCGCAT  CACGGCACGGCACGGCACGGCACATCTTCTTACAGTCACCATGCGAGCTG  CCGCACACAGGGCGCTTCGCGCGCCGCTCCTCGTCTCGGTGCAGGCAAGG  CAGGGAGCGCAACTTGCTCCCCAACAATGTCGGACATGGCCACGCTGCGC  GGCCCTCTCGACAGCGCCGACGCCAGCCGCAGGGGACGACGCAGATGCTG  CAGTGCGCCGCAGCGTACTGTACATGCCCTCCTCGCGGCAGCGAGCCCTC  GAAAAGGCCAAGACCATCAAGGCCGACGCCTATATCTTTGACCTCGAGGA  CGCCGTGTCGCCAGAGCAAAAAGAGCTTGCACGCCAACAGGCCGTCGCGG  CCGCGCGCGAGCAGGTCGAGACCAAGGCCTATGGCATGTCTGAAATCTGT  ATCCGGGTCAATGGGCGCACCACGCCCTGGTACGCGAGCGATGTCGAAGC  CGTAGCGACGAGCGGGGCCAACGCTGTGCTCCTTCCCAAGGCTGAGTCCC  GCGATGATGTGGCCGACCTCGTGGACAGGCTTGATGCAGCTGGCGCAAAA  CCCTCTATGGAGATTTGGTGCATGGTAGAGACACCGCGCGGCGTAAGCAA  TGTCGAAGAGCTCGCAGCTCATCCGCGCGTCACTGCACTTCTCATGGGTA  CTGTCGACCTTGCAAATGACTTGCGTTGTAAACCGATGGCTCCAGGACGC  TTTAACCTGCAATACGCGCTTCAGCGCTGCGTCATTGCTGCTCGCGCCGC  CGGCATTAGTGCCTTGGATGGTGTTTATGTTGATCTCCAAAACGAAGAGG  GCTTCATCGACGAGTGTCTCCAGGGTCGTGACCTTGGCTTTGATGGAAAA  ACGCTCATTCACCCCAAGACGGTGGCCCAAGCTAACGAATTCTTTTCTCC  CAGCGCAGCCGACATTGACCACGCCGAGAGAGTCATTGCGGCACACGAAG  ACGCCGTTCGCACCGGCTCAGGGGTCGCCACTGTCGACGGCAAGCTTGTT  GAGAACCTTCACTACCGTAATGCCAAGCGCACGCTTGCGATTGTTGAGCG  CATCAAGGCGCGTGCATGA |
|  |  |
| *ACC* | ATGATGCAGAACGAGAGCATCCCCGGCGCCGCGCAGGATGCCAAGCTCCG  CACGTTCGAGAACATGGAGGAGTACGTCAAGTCGCAGGGCGGCAAGCGCG  TAATCAAAAAGGTCCTCGCCGCCAACAACGGCATGGCCGTCGCCAAGCTT  CTCAAATCCATCCGGTCCTTTTGCTACTCGACCTTTGGCCGCGAAAACGA  GATCGAGGTCATCTGCATGGCCACCCCCGAGGATCTCGGCGCCAACGCCG  AGTACATCCGTGCCGCCGATCAGGTCGTGCACGTCCCCGGAGGCTCGAAC  GTGAACAACTACAACAACGTCAGCCTCATCGTGGAGATCGCCCAGCAGTA  CAAGGTCGACGCCGTCTGGGCCGGCTGGGGTCACGCCAGTGAGAACCCGG  TCCTCCCAGCCACTCTCTCCGAGCTCGGCATCGTTTTTGTGGGCCCCCCT  GCCGGCCCGATGAATGCCCTCGGTGACAAGATCATGAGCAGCATCGTCGC  GCAGTCGTGCGGCTGCCCCATGATCGCCTGGAACGGCTCCGACATCCGCG  TCAACTACAAGGAAGATGGCGGTGTCTCGGACGAGATCTTCGACTCGGCC  AACGTGCAGACCGTCGAGGACGCCAAGAAGCAGGTCGAGAAGATTGGCGT  CCCCGTCATGATCAAGGCCAGTGAAGGTGGTGGCGGCAAGGGTATCCGCC  TCGTCGACGACTACTCCAAGGTCGAAGCCTGCTTCCGTCAGGTCCAGAGC  GAGGTGCCCGGCAGCCCCATCTTCATCATGCGCCTCGCCGAGCGCGCCCG  CCATCTCGAGGTCCAGCTCCTCGCCGACGAGTACGGTAACGCCATTGCCC  TCAGCGGCCGCGACTGCTCCGTCCAGCGTCGCCACCAAAAGATTCTCGAG  GAGGGCCCGCCCGTGGCCGCCAAGCCCGAGGTCTGGAAGCAGATGGAGCA  CGCCGCCGTCAAGCTCGCCAAGGAGGTCGGCTACGTCAACGCCGGCACCG  TCGAGTACCTGTACGATGACAACGACAACTTCTTCTTCCTGGAGCTCAAC  CCTCGTCTCCAGGTCGAGCACCCGGTGACCGAGATGATCACCGGCACCAA  CGTGCCGGCTGCCCAGCTCCAGGTTGCCATGGGCATCCCCCTCAACCGCA  TCCCGGACGTGCGCCGCTTCTACGGCTGCGACGATCTCTTTGCGGACGAG  CCCATCGATTTTGACGGCGAGTACGCCGACGAGCCCACCACCACGAGCAT  TACTCGCGGCCACACCATTGCTGCCCGCATCACGGCCGAAAACCCCTTCA  ACGGCTTCCAGCCCACCATTGGCCAGATTAGCGAGATCAACTTCCGCAGC  TACCGCAACGTGTGGGGCTACTTTAGCGTTGACAGCTACGGCCGCGTCCA  CGAGTTTGCCGACTCGCAGATTGGCCACGTCTTTGCCTGGGGCGAGACCC  GCGAGGAGGCTCGCCGCAGCCTTGCCATGGCGCTCCACGACCTCTCGATC  CGCGGTGAGATTCGCACCACCATCGAGTACCTCAAGGATCTCATCGAGTC  CGAGGACTATGTCAACAACAAGTTTAATACCGCCTGGCTCGATGCCCGCA  TCAAGAGCAACATTGCCGTCTCCAAGATGGACCCGCTCACCATTGCTCTC  GTCGGTGGTGTCTGCACCGCGCACCGCGAGATCGCCGCGCGTGGCGCTGA  CTACATGAGCATGCTTGAGCGTGGCCAGCTCCCGCCCGTGCCCCTGCTCG  ACCAGGCGCACGCCTTTGAGCTCATCTACGAGGGCGTCAAGTACAAGCTC  AACGGCTGCCACACCGGCGAGAACACCTATCGCCTCTACTGCAACGGCAG  CCACGTCGACGCCGAGCTCCGCTGCCTCGCGGACGGTGGCTTCCTCGTGC  TCATTGGCGGCCGCTCTCACGTCGCCTACGTCAAGGAGGATGTTGGCGCC  CAGCGCTACACTATCGACGGCCAGACCTGCCTCTTTGAGGACGAGTATGA  CCCGACCCAGATGCGCGCCCAGATGGGTGGCAAGCTCCTCCGCTACCTCG  TCGAGGATGGTGCCTCGCTCGAGAAGGGCGACGGCTTTGCCGAGATTGAG  GTCATGAAAATGAACATGACCCTCTCGGCCCTCGAGGCCGGTACCATCAC  GCTGCACAAGCCCGAGGGTGCCGTCATGGAGCCCGGTGACATGATTTGCA  CCATGGAGCTCAAGGACCCCAGCAAGGTGCAAAAGGCCAAGCTCTTCGAG  GGCACCTTCCCCGCCCTTGGCGAGCCCTGGCCCCAGACTCTCCGCAACAT  GCCGCACCACACCCTCGAGCGCGCCCAGCGTCGCCTCGAGGCCGTCATGG  CCGGTTTCGCGATCGAGAATGAGGTCACTGTCGAGGCCCTCAAGAGCCTC  CGCGATGCCCTCCACAGCCCGCTCCTCCCCGTGCTTGAGATCGAGGGCAT  CGTCTCGCGCACCAAACACGCGCTTCCCAAAGCGCTCCTCAGCAAGGTCG  AGAGCCTCTGCCGCGAGCTCCGCTCCAAGGAGGAGCCCAGCCTCGACGAC  TCGGCGGCCTTTGCCGCTGCCGTGCTCTCGGCGGCCCAGGAGCACCCTCC  TGCTCAGGTTGGCGAGATCCTCTCGGTGGCCGAGTCGTACAAGGACGGCC  TCAGTATCCTCTACGCGCGCATCCTTGGCAAGCTTATCGCCAGCTTTGTC  GAGGTCGAGGCCAAGTTTGCCGAGCTCGAGAACTCGGACGCTGCCGACAA  GGATGACGTCCTCCAGGAGCTCCGCGCCCAGAACGCTGGCGACCTCCCCA  AGGTCCAGCGCTTTGCGCTCGCGCACCACTCGCGCAAGCCCCGCGACACC  CTCGTGCTCGCCATCCTCGAGGAGCTCGACACCCTGCAGCGTGGCACCGA  GACCCGCAGCGCCTCGCCCAAGCTTCGCTCCGAGTGTGCCAACATCATGC  AGGAGGTCGCCGCTCTCCAGGGCATCAAGACCACGGATGTGGCCCTCGAG  GCTCGCCAGTCGCTCATTGAGATGGAGCACTCGTACGAGGACCAGCTCAA  GTCTGTAACTGACAAGCTCAAGCGCGTCATGCAGGGCGATGAGTCCGCCC  GCGAGGAGCTCGTGCAGTCCACCGAGCCGGTTCTTGCTTACCTCATGGAC  ATTGTCTCGCGCTTCAACGAGAACCCGGCCGACCTTCGCCAGACTGCCCT  TCGCGTCTACGTGAGCCGCGTCTACGCTGCCTACAAGGTCGTTGATGCCT  CGACGAGCGAGCTGACCAAGAACAACCTTGCCTGCGACTTTACGTTCTTC  TCGGAGGCGACCGACTCTGTCCAGGTCGGCGGTGGCGGCTCGGGTCTCGC  GAACGTGTCTTCGTTCGAGGACCTCACCAAGGTGCTCAGCAACAACGGCG  ACTTTGACTTTGGCGCGACCAACGAGTCGAACCGCTCGGATGCCGAGACC  AGCAGCGTTGGCATGGACCTCGACATTGGCGGCGGCGCCGAGAGCAGCGC  CCCGACCTCGGGCCTCTCTGGCGGCAAGTCGATGCTTGCGCGCCAGGGCG  GCGACTTTAAGGCTCGCTCCGCCTCGGTGGAGGGCCCGGCCTCGTCCACG  GTCCCGCCGTACGTGAACCGCAAGGGCAAGCTCCTCTTTTTCGCCGACAT  GGCCGAGCTCGAGGAGAACCTTGCGTCCTCGATCAAGGACTTTGGCTCCG  ACACTTCGTCGCCCACGCCGCTCAACGTCGTGCATGTCATCTTTGGCACG  CTCGTCGACTCGGAGACTGATGTTTCGGCCAAGCTGTACGAGGTCGTGCA  GAAGAACAAGGTTGTGCTCAGCGAGAACCTCGTGCGCCGCATCACCTTTG  CCGTGGTGCGCTCCCAGTACGAGGAGGCTGACCGCCACGTGGCCATTGTT  GCCGGCCATGGTGGCCACTTCTTCACTTTCCGCAACAGCTCCGGCTACGA  GGAGGACCGTCTCGTGCGCAACATTGAGACGCCGCTGGCCTTCCAGCTCG  ACCTCGAGCGCATGTCGAACTTCAACATTCGCATGGTGCCCATCGGTCGC  AGCATGAGCCGCTCGCAGGCTGTGCACGTGTACGAGGCGACGCCCAAGGC  CAACGCCGCCGGCAAGGTTGTGGGCATGCGCCGCTTCTTTGTGCGCGCTC  TCGTCCGCGATGCCGAGCGCGTCAAGCTCGACGTCGGCACCTTCGATGCC  TACCCTGGCCCGGAGCGTGTCTTCGTGCAGGCCCTGCGCGCCCTCGAGTC  CGTCCAGGACGACTCGAAGACCAAGGCCCGCAAGAACCACATCTTCATGA  ACGTGCTCAGCGACTCGGCCACCGTCGACGCTGGCTACGTCGAGGGCATC  ATCCGCACGCTCCACCGCCGCTACGCCAAGCGCCTGATTTCGCAGAACGT  CGAGGAGTTTGAGATCCGCGTCAACGCTGTTCTCGCCGAGGGCGCGCCGA  GCATGCCCATTCGCGTCATCGCCTCGAACCCGACCGGCTTTGCCCTCAAC  ATCGACACCTACGTCGAGGCCTCGGACCCTTCGGGCTCGCAGGATGCCAT  GTACTACTCGATCTCCGAGGGCGACGGCGGCCTTGGCGGCGCCCTTGCCG  CCATGGGCCTCGGTGCCGGTGAGGCCGACGTCAACGCCGGCTCCACGGAC  AGCACGGGCTCGCTCCACGGCAAGCCCCTGCACACGCCGTACCCGGTCTC  GGACGAGTTCGACGAGCGCCGTGCCCGCGCCCGCGCTGCCAGCACGACCT  TTGCGTACGACTTTCCGGATCTCTTCCGCAAGTCGCTCGAGTTTGCCTGG  CGCGAGCACCTCAGCACGACCGGCACCAAGGAGAACATGCCGCCCCGCAA  GAGCCTCGTCGAGGCCGAGGAGCTCGTCCTCGACGACGAGTTCGAGGCTG  GCGATGCCGTCGACCCGGTCAACGCCCCCCGCCTCTGCCGCGTGGACCGC  AAGGCCGGCCGCAACCCGATCGGCATGGTCGCGTGGCGCTTCTTCATGCG  CACCCCGCAGTACCCGCGTGGCCGCGAGGTCGTGGTCATTGCCAACGACA  TCACCGTCAAGGCTGGCTCCTTTGGTACCCGCGAGGACATGCTCTTTGAC  CAGGCCTCCAAGTACGCGCGCCTGAATGGGCTCCCCCGCCTGTACATTGC  CGCGAACTCTGGCGCCCGCATCGGCATGGCCGACGAGGTCAAGCGTGCCT  TCCAGGTCAAGTGGATTAACGAGGCCGACCCGACCAAGGGCTACGAGTAC  ATTTACGTGAACGAGGATACCTTCAATCAGCTCGGCCCCGACGGCCGCAA  GAGCTTGCTCGCTGAGAAGGTCGAGGGCACGGACCACTTCCGCATCAACG  CCATCGTTGGTGAGTCCCCGGACCTTGGTGTGGAGAACCTCCGCGGCTCG  GGCACCATTGCCGGCGAGACGGCGCGTGCCTACGAGGAGTCCTTTACCCT  CTCGTACGTGTCGGGCCGCTCGGTCGGTATTGGCGCCTACCTCGTCCGCC  TTGGCCAGCGCATTGTGCAAAAGGGCAAGAACGCCCCGATCCTGCTCACC  GGCTACCAGGCGCTCAACTCGCTCATGGGCCGCGAGGTGTACACCTCGAA  CTTGCAGCTCGGTGGCACCAAGGTGATGTTCGCCAACGGTGTCTCGCACC  AGAGCGTCCGCCACGACCTCGAGGGTGTGGCCTCCATGGTCAAGTGGCTC  TCGTACGTGCCGGAGCGCCGCGGCGCCCCGCTCCCGCTCACGGCCCTGGT  CTCGGGTGACCGCATCGACCGCGACGTCGAGGTGCACCCGCGCGACCTCG  GCTCGGACTACGACCCGCGCACCCTGCTCACCGGTCTCTCCAAGGAGGAC  GGCAGCTTCCTCGGCGGCTTCTTTGACAAGGACTCGTTTACCGAGACGCT  CTCCGGCTGGGCTCGCACCGTCATTGCCGGCCGCGCTCGCCTGGGCAAGC  TGCCCATGGGTGTCATCATCTCGGAGATTCGCACCGTCGAGGCGCGCGCG  CCCGCTGACCCTGCGGCGCCCGAGTCCCAGGAGCTCATCTGGAACCAGGC  CGGTCAGGTGTGGTTCCCGGACTCCTCGTACAAGACGGCGCAGGCCATCA  ACGACTTCAACCGCGAGGGTCTCCCGCTCATGCTCTTTGCCAACTGGCGT  GGCTTCTCGGGCGGTACCCGCGACATGTTCGACCAGATTGTCAAGTTTGG  TGCCTACATTGTCGACGCCCTCGTCGCGTACAAGCAGCCTGTCTTCGTGT  ACATCCCGCCCTTTGGCGAGCTGCGCGGCGGCGCTTGGGTCGTGGTCGAC  GAGACCATCAACCCGTCCATGATGGAGATGTACGCCGACACCGACGCGCG  CGGTGGTGTCCTCGAGCCGGCCGGTGTCGTCTCGATCAAGTACCGTGCCA  AGGATGTCCTCGCCACGGCGCACCGCGTCGACGAGAAGCTCAAGGGCATG  GTCGAGAAGCTCAAGGCCGCCGCCCCGGACTCTGCCGAGGCCGCGGACCT  GAAGAAGGCCATTGCCGAGCGCGAGAAGCTCCTCATGCCCATCTTCAAGC  AGATTGCCGTGCACTTTGGCGACCTCCACGATCGCCCGGGCCGCATGCAG  GCCAAGGGTGTCATCCGCAGCGTGGTCCCCTGGAGCAACTCGCGCCGCCA  CTTTTACAACCGCCTGCGCCGCCGTCTTGCCGAGCTCGACGCCGTCGCCA  AGATCGACGAGGTCGTTGCCCTTTCGGACGACCTCGGTGCCCCGAGCTCG  CAGCCGCTCGAGATTCTCGAGAGCGTCTTCAACGCCTCGTGCGACTCGAG  CGTCCCGGACTGGAACAACGATAAGACGGTCCACGAGTGGCTTGTCTCCG  AGCAGGGCCAGCAGGCCGTGTCCAAGCACCTCGCCGGCATCAAGGCGGAC  GCTATCTCGAACAAGGTCACTTCCCTCGGCATGGAGGACCCCAAGGCCAT  CCTCAAGGGCCTCATGGGCGTCATCTCTAAGCTCCGCGACGAGGAGCGTG  AGGAGGAGCGCGCCGCTCTCGTCAACCTTCTGCGCAAGGGCTCGCTCCTG  CTCAACTAA |

**Table S2 Primers used in this study**

| **Purpose** | **Primer** | **DNA sequence (5'–3')** | **Length (bp)** |
| --- | --- | --- | --- |
| For developing a LacZ reporter system | lac-Fw | GGGGTACCATGACCATGATTACGGAT (*Kpn*I) | 3,075  458  351  1,075  614  869  473  411  318  1,269  7,059  869  473  2,611 |
|  | lac-Rev | ATAAGAATGCGGCCGCTTATTTTTGACACCAGAC (*Not*I) |  |
|  | EF1αp-Fw | CCGGAATTCACTCGTCCCAGGGTGGTT (*Eco*RI) |  |
|  | EF1αp-Rev | GGGGTACCGCAAAGTAGGCGGTAATA (*Kpn*I) |  |
|  | EF1αt-Fw | ATAAGAATGCGGCCGCGTTGGCTTCAACGTCAAG (*Not*I) |  |
|  | EF1αt-Rev | GCTCTAGAGAAGCGGCCAAGGGGCGG (*Xba*I) |  |
|  | ubip-Fw | CCGGAATTCCAGTCACTTTGACGCATT (*Eco*RI) |  |
|  | ubip-Rev | GGGGTACCTCTTGCTGCGTGTGAATC (*Kpn*I) |  |
|  | ubit-Fw | ATAAGAATGCGGCCGCCCAAGGCCAAGTCGGACT (*Not*I) |  |
|  | ubit-Rev | GCTCTAGATCGGTACCACCGCGTAAT (*Xba*I) |  |
|  | ccg1p-Fw | CCGGAATTCTCGACGGTATCGATAAGC (*Eco*RI) |  |
|  | ccg1p-Rev | GGGGTACCTTTGGTTGATGTGAGGGG (*Kpn*I) |  |
|  | ccg1t-Fw | ATAAGAATGCGGCCGCCTCCATGGCCTCCTCGAC (*Not*I) |  |
|  | ccg1t-Rev | GCTCTAGACGGCTGCAACAAGATTGT (*Xba*I) |  |
|  | TEF1p-Fw | CCGGAATTCTTTTCTCTTTCAGTGACC (*Eco*RI) |  |
|  | TEF1p-Rev | GGGGTACCCCAGGCCAGGGTGTTGTC (*Kpn*I) |  |
|  | CYC1t-Fw | ATAAGAATGCGGCCGCTCATGTAATTAGTTATGT (*Not*I) |  |
|  | CYC1t-Rev | GCTCTAGAGCAAATTAAAGCCTTCGA (*Xba*I) |  |
| For ACL and ACC overexpression | ACL-Fw | GGGGTACCATGGGGCCTAGGCCTACC (*Kpn*I) |  |
|  | ACL-Rev | ATAAGAATGCGGCCGCTCATGCACGCGCCTTGAT (*Not*I) |  |
|  | ACC-Fw | ATGATGCAGAACGAGAGCAT |  |
|  | ACC-Rev  ccg1pS-Fw  ccg1pS-Rev  ccg1tS-Fw  ccg1tS-Rev  ACLS-Fw  ACLS-Rev | TTAGTTGAGCAGGAGCGAGC  AGAGAGGCTGAAGCATCGATGAATTCTCGACGGTATCGATAAGC (*Eco*RI)  ATGCTCTCGTTCTGCATCATTTTGGTTGATGTGAGGGG  GCTCGCTCCTGCTCAACTAACTCCATGGCCTCCTCGAC  GACGGCCGGCTGGGCCACGTCGGCTGCAACAAGATTGT  AGAGAGGCTGAAGCATCGATGAATTCTCGACGGTATCGATAAGC (*Eco*RI)  AAGCTTATCGATACCGTCGACGGCTGCAACAAGATTGT |  |
| For verification of transformants | AOX-Fw | GACTGGTTCCAATTGACAAGC | 130  150  109 |
| For qRT-PCR | AOX-Rev  actin-QP-Fw  actin-QP-Rev  acl-QP-Fw  acl-QP-Rev  acc-QP-Fw  acc-QP-Rev | GCAAATGGCATTCTGACATCC  GCGACATCAAGGAGAAGC  GAAGGACGGCTGGAAGAG  CAGGGAGCGCAACTTGCT  GAGGGCTCGCTGCCGCGA  TCAAGAGCAACATTGCCG  CTCATGTAGTCAGCGCCA |  |

Underlining: restriction endonuclease site
